# Supplementary material for: The association between HIV diagnosis disclosure and adherence to anti-retroviral therapy among adolescents living with HIV in Sub-Saharan Africa: A systematic review and meta-analysis
Source: PLoS One. 2023 May 11;18(5):e0285571. doi: 10.1371/journal.pone.0285571 (PMC10174542; doi:10.1371/journal.pone.0285571)
Supplement: S2 Table — (DOCX) [file pone.0285571.s002.docx]

| Supplementary Table S2: Search strings used for a comprehensive search in databases |
| --- |
| PubMed: (((africa south of the sahara[MeSH Terms]) AND ((adolescent[MeSH Terms]) OR adult children[MeSH Terms])) AND ((((((((medication adherence[MeSH Terms]) OR patient compliance[MeSH Terms]) OR anti-retroviral agents[MeSH Terms]) OR antiretroviral therapy, highly active[MeSH Terms]) OR adherence to antiretroviral therapy[Title/Abstract]) OR adherence to highly active antiretroviral therapy[Title/Abstract]) OR adherence to haart[Title/Abstract]))) AND ((((((((truth disclosure[MeSH Terms]) OR self disclosure[MeSH Terms]) OR disclosure) OR status disclosure[Title/Abstract]) OR HIV serostatus disclosure[Title/Abstract]) OR HIV status disclosure[Title/Abstract]) OR HIV diagnos*s disclosure)) |
|  |
| Ovid(MEDLINE):1 (disclosure.mp. OR self disclosure.mp.) AND ( adherence.mp. OR compliance.mp. OR Anti-Retroviral Agents/ or Antiretroviral Therapy, Highly Active/ or antiretroviral therapy.mp. or Anti-HIV Agents/) OR ART.mp. OR HAART.mp.) AND (adolescent*1.mp. OR teenage*.mp.) AND (Africa, Western/ or South Africa/ or Africa, Eastern/ or africa.mp. or "Africa South of the Sahara"/ or Africa, Central/ or Africa/ or Africa, Southern/) |
|  |
| EMBASE: ('interpersonal communication'/exp OR 'self disclosure'/exp OR 'disclosure'/exp OR 'status disclosure' OR 'hiv serostatus disclosure' OR 'hiv diagnos$s disclosure') AND ('medication compliance'/exp OR 'adherence to antiretroviral therapy' OR 'adherence to highly active antiretroviral therapy' OR 'adherence to haart' OR 'compliance to art' OR 'adherence to art' OR 'compliance to art drugs' OR 'hiv drug adherence' OR 'antiretroviral therapy') AND ('adolescent'/exp OR 'adult child' OR 'teenager') AND ('africa south of the sahara'/exp) |
|  |
| HINARI:((SubjectTerms:(disclosure)) OR (HIV status disclosure) OR (HIV diagnosis disclosure)) AND ((SubjectTerms:(adherence)) OR (adherence to ART) OR (antiretroviral therapy)) AND ((SubjectTerms:(adolesent)) OR (SubjectTerms:(adult children)) OR (SubjectTerms:(teenager))) AND ((SubjectTerms:(africa south of the sahara)) OR (SubjectTerms:(sub saharan africa)) OR (SubjectTerms:(africa, western)) OR (SubjectTerms:(africa, central)) OR (SubjectTerms:(africa, eastern)) OR (SubjectTerms:(africa, southern))) |
|  |
| Google Scholar: (hiv-disclosure adherence antiretroviral therapy "adolescents living with HIV" ) (Sub?suharan Africa OR Angola OR Benin OR Botswana OR Burkina Faso OR Burundi OR Cameroon OR Cape Verde OR Central Africa OR Central African Republic OR Chad OR Comoros OR Congo OR Cote d'Ivoire OR Democratic Republic Congo OR Djibouti OR Equatorial Guinea OR Eritrea OR Eswatini OR Ethiopia OR Gabon OR Gambia OR Ghana OR Guinea OR Guinea-Bissau OR Kenya OR Lesotho OR Liberia OR Madagascar OR Malawi OR Mali OR Mayotte OR Mozambique OR Namibia OR Niger OR Nigeria OR Rwanda OR Sahel OR Senegal OR Sierra Leone OR Somalia OR South Africa OR South Sudan OR Sudan OR Tanzania OR Togo OR Uganda OR Zambia OR Zimbabwe) |
