# Supplementary material for: The association between HIV diagnosis disclosure and adherence to anti-retroviral therapy among adolescents living with HIV in Sub-Saharan Africa: A systematic review and meta-analysis
Source: PLoS One. 2023 May 11;18(5):e0285571. doi: 10.1371/journal.pone.0285571 (PMC10174542; doi:10.1371/journal.pone.0285571)
Supplement: S3 Table — (DOCX) [file pone.0285571.s003.docx]

Supplementary Table S3. Study quality assessment results for case-control, Cohort and Cross-sectional studies

| Author, year | Study design | Q1 | Q2 | Q3 | Q4 | Q5 | Q6 | Q7 | Q8 | Q9 | Q10 | Q11 | Risk of bias (RoB) |
| --- | --- | --- | --- | --- | --- | --- | --- | --- | --- | --- | --- | --- | --- |
| Arage 2014 | Cross-sectional | Yes | Yes | No | Yes | Yes | Yes | No | Yes |  |  |  | Moderate RoB |
| Biressaw, 2013 | Cross-sectional | No | Yes | No | Yes | Yes | Yes | Yes | Yes |  |  |  | Moderate RoB |
| Cluver, 2015 | Cross-sectional | Yes | Yes | Yes | Yes | Yes | Yes | Yes | yes |  |  |  | Low risk of bias |
| Dachew, 2014 | Cross-sectional | Yes | Yes | No | Yes | Yes | Yes | No | Yes |  |  |  | Moderate RoB |
| Montalto, 2017 | Retrospective cohort | unclear | Yes | Yes | Yes | Yes | Yes | Yes | Yes | Yes | Yes | Yes | Low RoB |
| Nabukeera-Barungi, 2007 | Cross-sectional | Yes | Yes | Yes | Yes | Yes | Yes | Yes | Yes |  |  |  | Low risk of bias |
| Orji, 2018 | Cross-sectional | No | Yes | No | No | No | No | No | No |  |  |  | High RoB |
| Tjituka, 2018 | Cross-sectional | Yes | Yes | Yes | Yes | No | No | Yes | No |  |  |  | Moderate RoB |
| Kimanthi, 2016 | Cross-sectional | Yes | Yes | Yes | Yes | No | No | Yes | No |  |  |  | Moderate RoB |
| Fikadu, 2013 | Cross sectional | Yes | Yes | Yes | Yes | Yes | Yes | No | Yes |  |  |  | Low RoB |
| Mengesha, 2022 | Case-control | Yes | No | Yes | Yes | Yes | Yes | Yes | No | Yes | Yes | - | Low RoB |
| Newman, 2016 | Cohort | Yes | Yes | Yes | Yes | Yes | No | No | Yes | No | Yes | Yes | Moderate RoB |
| Edun 2022 | Cohort | Yes | Yes | Yes | Yes | Yes | No | Yes | Yes | Yes | No | Yes | Low RoB |
| Kairania et.al 2022 | Cross-sectional | Yes | Yes | Yes | Yes | Yes | Yes | No | Yes |  |  |  | Low RoB |

Interpretation: Q1-Q11 represents the items used for the quality assessment

Cross-sectional (total items: 8): 7-8, low RoB; 4-6, moderate RoB; 0-3, high RoB.

Case-control (total items: 10): 8-10, low RoB; 7-10, moderate RoB; 0-4, high RoB.

Cohort (total items: 11): 9-11, low RoB; 5-8, moderate RoB; 0-4, high RoB.

The critical appraisal tools can be accessed at: https://jbi.global/critical-appraisal-tools
