# Supplementary material for: The association between HIV diagnosis disclosure and adherence to anti-retroviral therapy among adolescents living with HIV in Sub-Saharan Africa: A systematic review and meta-analysis
Source: PLoS One. 2023 May 11;18(5):e0285571. doi: 10.1371/journal.pone.0285571 (PMC10174542; doi:10.1371/journal.pone.0285571)
Supplement: S4 Table — (DOCX) [file pone.0285571.s004.docx]

Supplementary Table S4: Reasons for excluded primary studies

| N^o^ | Author, year | Title | Reference URL | Main reason for exclusion |
| --- | --- | --- | --- | --- |
|  | Abera 2015 | Factors influencing adherence to antiretroviral therapy among people living with HIV/AIDS at ART Clinic in Jimma University teaching hospital, Southwest Ethiopia | <https://link.springer.com/article/10.1007/s10461-017-1944-x> | Adult population |
|  | Abiodun 2016 | Predictors of adherence to antiretroviral therapy among people living with HIV and AIDS at the Quthing District Hospital and Villa-Maria Health Centre, Lesotho | [Abiodun_Oluwasola_Afolabi_2016.pdf (regroup-production.s3.amazonaws.com)](https://regroup-production.s3.amazonaws.com/documents/ReviewReference/199215168/Abiodun_Oluwasola_Afolabi_2016.pdf?AWSAccessKeyId=AKIAJBZQODCMKJA4H7DA&Expires=1622902438&Signature=EsdMBL59ix8DTqiQ%2FWXyXr33Vwg%3D) | Adult population |
|  | Achilla 2010 | Disclosure of HIV status to sexual partners amongst people who receive antiretroviral treatment in Kampala, Uganda | <http://hdl.handle.net/11394/2814> | Qualitative study |
|  | Ahonkhai 2016 | Age matters: increased risk of inconsistent HIV care and viremia among adolescents and young adults on antiretroviral therapy in Nigeria | <https://pubmed.ncbi.nlm.nih.gov/27329680/> | Outcome of interest not available |
|  | Ajong 2018 | Quantification and factors associated with HIV-related stigma among persons living with HIV/AIDS on antiretroviral therapy at the HIV-day care unit of the Bamenda Regional Hospital, North West Region of Cameroon | <https://doi.org/10.1186/s12992-018-0374-5> | Outcome of interest not available and adult population |
|  | Anies 2016 | Barriers to antiretroviral therapy adherence for HIV-positive adolescents in South Africa | <https://www.hindawi.com/journals/art/2017/5192516/> | Qualitative study |
|  | Arrive 2012 | HIV status disclosure and retention in care in HIV-infected adolescents on antiretroviral therapy (ART) in West Africa | - DOI: [10.1371/journal.pone.0033690](https://doi.org/10.1371/journal.pone.0033690) | Outcome of interest not available |
|  | Beima-Sofie 2017 | Pediatric HIV Disclosure Intervention Improves Knowledge and Clinical Outcomes in HIV-Infected Children in Namibia | Doi: 10.1097/QAI.0000000000001290 | Disclosure was an outcome not exposure variable |
|  | Bikaako-Kajura 2006 | Disclosure of HIV status and adherence to daily drug regimens among HIV-infected children in Uganda | <https://doi.org/10.1007/s10461-006-9141-3> | Qualitative study |
|  | Biru 2017 | Caregiver-reported antiretroviral therapy non-adherence during the first week and after a month of treatment initiation among children diagnosed with HIV in Ethiopia | <https://doi.org/10.1080/09540121.2016.1257098> | disclosure was not reported as exposure |
|  | Breger 2017 | Self-disclosure of HIV status, disclosure counseling, and retention in HIV care in Cameroon | <https://doi.org/10.1080/09540121.2016.1271390> | Outcome of interest not available |
|  | Buma 2015 | The Influence of HIV-Status Disclosure on Adherence, Immunological and Virological Outcomes among HIV-Infected Patients Started on Antiretroviral Therapy in Dar-es-Salaam, Tanzania | http://dx.doi. org/10.16966/2380-5536.111 | Adult population |
|  | Charles 2018 | Actors Associated with Antiretroviral Therapy Adherence among Adolescent Living with HIV: A Case of Kigoma Region | <http://repository.out.ac.tz/id/eprint/2349> | disclosure was not reported as exposure |
|  | Cluver 2018 | Multitype violence exposures and adolescent antiretroviral nonadherence in South Africa | [http://10.1097/QAD.0000000000001795](http://10.0.4.73/QAD.0000000000001795) | disclosure was not reported as exposure |
|  | Cluver 2018 | Stacking the odds for adolescent survival: health service factors associated with full retention in care and adherence amongst adolescents living with HIV in South Africa | [http://10.1002/jia2.25176](http://10.0.3.234/jia2.25176) | Outcome of interest not available |
|  | Denison 2018 | Factors Related to Incomplete Adherence to Antiretroviral Therapy among Adolescents Attending Three HIV Clinics in the Copperbelt, Zambia | <https://doi.org/10.1007/s10461-017-1944-x> | Outcome of interest not available |
|  | Filiatreau 2020 | Correlates of ART Use Among Newly Diagnosed HIV Positive Adolescent Girls and Young Women Enrolled in HPTN 068 | [http://10.1007/s10461-020-02817-1](http://10.0.3.239/s10461-020-02817-1) | Wrong patient population  (The study regressed for current ART use versus non-use not for adherence) |
|  | Firdu 2017 | HIV-infected adolescents have low adherence to antiretroviral therapy: A cross-sectional study in Addis Ababa, Ethiopia | <http://www.panafrican-med-journal.com/content/article/27/80/full/> | Disclosure was not reported as exposure (almost all were of their HIV status, 90.1%) |
|  | Fokam 2017 | Immuno-virological response and associated factors amongst HIV-1 vertically infected adolescents in Yaounde-Cameroon | [https://doi.org/10.1371/ journal.pone.0187566](https://doi.org/10.1371/%20journal.pone.0187566) | Disclosure was not available as an exposure |
|  | Gross 2015 | Factors associated with self-reported adherence among adolescents on antiretroviral therapy in Zimbabwe | <https://doi.org/10.1080/09540121.2014.969676> | disclosure was not reported as exposure and aware of their HIV status |
|  | Haberer 2011 | Excellent adherence to antiretrovirals in HIV+ Zambian children is compromised by disrupted routine, HIV Nondisclosure, and Paradoxical Income Effects | [http://10.1371/journal.pone.0018505](http://10.0.5.91/journal.pone.0018505) | Outcome of interest not available |
|  | Idindil 2012 | A case-control study of factors associated with non-adherent to antiretroviral therapy among HIV infected people in Pwani Region, eastern Tanzania | <http://dx.doi.org/10.4314/thrb.v14i3.6> | Wrong patient population |
|  | Jones 2013 | Determinants of engagement in HIV treatment and care among Zambians new to antiretroviral therapy | [http://10.1016/j.jana.2012.06.009](http://10.0.3.248/j.jana.2012.06.009) | Adult population |
|  | Kabogo 2018 | Evidence of reduced treatment adherence among HIV infected paediatric and adolescent populations in Nairobi at the onset of the UNAIDS Universal Test and Treat Program | <https://bmcresnotes.biomedcentral.com/articles/10.1186/s13104-018-3205-0> | disclosure was not reported as exposure |
|  | Kim 2017 | High self-reported non-adherence to antiretroviral therapy amongst adolescents living with HIV in Malawi: barriers and associated factors | <https://doi.org/10.7448/IAS.20.1.21437> | Outcome of interest not available |
|  | Martelli 2019 | Adherence to antiretroviral treatment among children and adolescents in Tanzania: Comparison between pill count and viral load outcomes in a rural context of Mwanza region | <https://doi.org/10.1371/journal.pone.0214014> | disclosure was not reported as exposure and outcome of interest was not available |
|  | Mudzviti 2015 | Adherence assessment techniques in adolescents receiving a protease inhibitor based antiretroviral therapy treatment regimen in a resource limited setting | regist2.virology-education.com/2015/9INTEREST/46_Mudzviti.pdf | Full text not available |
|  | Nabukeera-Barungi 2015 | Adherence to antiretroviral therapy and retention in care for adolescents living with HIV from 10 districts in Uganda | <https://www.ncbi.nlm.nih.gov/pmc/articles/PMC4647509/> | Disclosure was not reported as exposure |
|  | Nabunya 2020 | The role of family factors in antiretroviral therapy (ART) adherence self-efficacy among HIV-infected adolescents in southern Uganda | <https://doi.org/10.1186/s12889-020-8361-1> | Outcome of interest not available |
|  | Namoomba 2019 | Factors Influencing Adherence to Antiretroviral Therapy among HIV Positive Adolescents at Adult Infectious Diseases Center in Lusaka, Zambia | DOI: [10.4236/ojn.2019.94040](https://doi.org/10.4236/ojn.2019.94040) | disclosure was not reported as exposure |
|  | Naomi 2018 | Determinants of Adherence to Antiretroviral Among HIV Positive Adolescents at Comprehensive Care Clinic, Gertrude’s Children Hospital, Nairobi, Kenya | [http://doi: 10.11648/j.ajns.20180701.13](http://doi:%2010.11648/j.ajns.20180701.13) | Disclosure was not reported as exposure (only adolescents who know of their HIV positive status are included in the study.) |
|  | Natukunda 2017 | Beyond clinical trials: Cross-sectional associations of combination antiretroviral therapy with reports of multiple symptoms and non-adherence among adolescents in South Africa | [http://10.7196/SAMJ.2017.v107i11.12405](http://10.0.28.28/SAMJ.2017.v107i11.12405) | Wrong comparator  (not able to compute odds ratio for adherence from the data provided) |
|  | Ndayikeje 2018 | Predictors of poor adherence among children and adolescents on ART for at least 1year in Kigali Pediatric Centre of Excellence, Rwanda | [Predictors of poor adherence among children and adolescents on ART for at least 1year in Kigali Pediatric Centre of Excellence, Rwanda. \| American Academy of Pediatrics (aappublications.org)](https://pediatrics.aappublications.org/content/142/1_MeetingAbstract/546) | Wrong comparator |
|  | Ndiaye 2014 | A study on the barriers to Anti-retroviral Therapy adherence among Human Immunodeficiency Virus infected adolescents in Gaborone (Botswana) | <http://hdl.handle.net10539/13628> | disclosure was not reported as exposure |
|  | Ngeno 2019 | Disclosure and clinical outcomes among young adolescents living with HIV in Kenya | <https://doi.org/10.1016/j.jadohealth.2018.08.013> | Outcome of interest not available |
|  | Nichols 2019 | High prevalence of non-adherence to antiretroviral therapy among undisclosed HIV-infected children in Ghana | [http://10.1080/09540121.2018.1524113](http://10.0.4.56/09540121.2018.1524113) | All study subjects were unaware of their HIV status (wrong comparator) |
|  | Ojwang 2016 | Loss to follow-up among youth accessing outpatient HIV care and treatment services in Kisumu, Kenya | <https://doi.org/10.1080/09540121.2015.1110234> | Outcome of interest not available |
|  | Okatch 2020 | Trends in HIV Treatment Adherence Before and After HIV Status Disclosure to Adolescents in Botswana | [http://10.1016/j.jadohealth.2020.02.023](http://10.0.3.248/j.jadohealth.2020.02.023) | measure of effect size not reported |
|  | Ramos 2018 | Modality of Primary HIV Disclosure and Association with Mental Health, Stigma, and Antiretroviral Therapy Adherence in Tanzanian Youth Living with HIV | - DOI: [10.1089/apc.2017.0196](https://doi.org/10.1089/apc.2017.0196) | All study participants were aware of their HIV status |
|  | Revegue, 2020 (preprint) | 24-month clinical, immune-virological outcomes and HIV status disclosure in adolescents living with perinatally-acquired HIV in the COHADO cohort, in Togo and Côte d’Ivoire, 2015-2017 | Doi:10.21203/rs.2.15733/v3 | Outcome of interest not available |
|  | Sithole 2018 | Virological failure among adolescents on ART, Harare City, 2017- a case-control study | <https://doi.org/10.1186/s12879-018-3372-6> | Outcome of interest not available |
|  | Ugwu 2013 | Factors influencing adherence to paediatric antiretroviral therapy in Portharcourt, South- South Nigeria | <https://doi.org/10.11604/pamj.2013.16.30.1877> | Wrong patient population |
|  | Woollett 2016 | Adolescents living with HIV: Emerging issues in public health in South Africa | <https://doi.org/10.1007/978-3-319-29936-5_4> | A review (book chapter) |
|  | Orji 2018 | Status Disclosure in HIV Infected Children in Abakaliki, Ebonyi State, Southeast, Nigeria | https://doi.org/10.3126/jnps.v37i3.18730 | Poor quality |
