# Supplementary material for: The association between HIV diagnosis disclosure and adherence to anti-retroviral therapy among adolescents living with HIV in Sub-Saharan Africa: A systematic review and meta-analysis
Source: PLoS One. 2023 May 11;18(5):e0285571. doi: 10.1371/journal.pone.0285571 (PMC10174542; doi:10.1371/journal.pone.0285571)
Supplement: S5 Table — (DOCX) [file pone.0285571.s005.docx]

Supplementary Table S5. Sensitivity analysis of primary studies included in the meta-analysis of the association between disclosure and adherence to anti-retroviral treatment

| **Excluded Study** | **Pooled Effect size** | **95% CI** | I^2^ | **P-value** |
| --- | --- | --- | --- | --- |
| Arage 2014 | 1.49 | (0.75, 2.94) | 95.3% | <0.0001 |
| Cluver 2015 | 1.53 | (0.76, 3.07) | 95.6% | <0.0001 |
| Biressaw 2013 | 1.74 | (0.84, 3.62) | 96% | <0.0001 |
| Dachew 2014 | 1.88 | (1.21, 2.94) | 79.8% | <0.0001 |
| Nabukeera 2007 (a) | 1.65 | (0.81, 3.37) | 95.9% | <0.0001 |
| Nabukeera 2007b | 1.51 | (0.75, 3.03) | 95.80% | <0.0001 |
| Tjituka 2018 (UnPub) | 1.60 | (0.81, 3.14) | 95.6% | <0.0001 |
| Fikadu 2013 (UnPub) | 1.60 | (0.81, 3.14) | 95.6% | <0.0001 |
| Kimanthi 2016 (UnPub) | 1.60 | (0.81, 3.14) | 95.6% | <0.0001 |
| Mengesha 2022 | 1.51 | (0.75, 3.03) | 95.7% | <0.0001 |
| Newman 2016 | 1.66 | (0.82, 3.36) | 96.0% | <0.0001 |
| Edun 2022 | 1.67 | (0.74, 3.80) | 95.5% | <0.0001 |
| Kairania 2022 | 1.63 | (0.79, 3.39) | 95.8% | <0.0001 |
